# Comprehensive evaluation of fruit quality for premium Wangmo *Castanea mollissima* plants

**DOI:** 10.1371/journal.pone.0295691

**Published:** 2023-12-12

**Authors:** Li Long, Lingling Lv, Jie Qiu, Dongchan Sun, Shangfeng Wei, Xianqin Wan, Chao Gao

**Affiliations:** Institute for Forest Resources and Environment of Guizhou, Key Laboratory of Forest Cultivation in Plateau Mountain of Guizhou Province, College of Forestry, Guizhou University, Guiyang, 550025, China; Mount Kenya University College of Health Sciences / Kenyatta University, KENYA

## Abstract

In this study, the fruit phenotype and quality of 32 superior Wangmo *Castanea mollissima* plants (designated GM1 to GM32) were examined to identify the trait characteristics of different cluster groups and germplasms with excellent comprehensive performance. The goal was to provide a theoretical basis and research foundation for collecting high-quality germplasm resources and breeding superior cultivars of Wangmo *C*. *mollissima*. Ten fruit phenotypic traits and 13 quality traits were measured and analyzed in these 32 superior Wangmo *C*. *mollissima* plants. Cluster analysis and principal component analysis (PCA) were used to perform a comprehensive evaluation. Extremely significant positive correlations (*P*<0.01) were observed for 15 pairs of fruit phenotypic and quality traits, and significant positive correlations (*P*<0.05) were observed for 16 pairs of traits. Highly significant negative correlations (*P*<0.01) were observed for 4 pairs of fruit phenotypic and quality traits, and significant negative correlations (*P*<0.05) were observed for 15 pairs. The plants were divided into three groups by cluster analysis: the first group had large fruits and good fruit quality, the second group had small fruits and poor fruit quality, and the third group had medium-sized fruits with a high starch content. Four principal components were extracted from the 23 traits by PCA, contributing 76.23% of the variance. The ten plants with the highest comprehensive quality were GM32, GM31, GM29, GM1, GM8, GM17, GM10, GM30, GM3 and GM28. The results of this study provide a reference for the development and utilization of Wangmo *C*. *mollissima* germplasm resources.

## Introduction

*Castanea mollissima* Blume (chestnut) belongs to the Fagaceae family and the *Castanea* Mill genus. It is an ancient species and was one of the first domesticated fruit trees in China. Chestnut has been cultivated for more than 2000 years [1,2] and has important economic and ecological value. *C*. *mollissima* (chestnut) is typically used in cooking after boiling, roasting, frying, or other culinary treatments. It is rich in a variety of nutrients that are beneficial to human health, such as starch, soluble sugar, soluble tannin-binding proteins and acids [2]. Chestnuts can not only provide energy for the human body but also maintain a certain osmotic pressure and have important nutritional benefits. In addition, *C*. *mollissima* contains important nutritional elements, such as potassium (K), calcium (Ca), magnesium (Mg), manganese (Mn), iron (Fe), and zinc (Zn), which play a vital role in maintaining homeostasis in the human body [3–5].

Wangmo County is a traditional production area for chestnuts in China and is renowned as the “Famous County of Chinese Chestnuts” and a chestnut “geographical indication” county. According to analyses conducted by Chinese authorities, Wangmo *C*. *mollissima* is rich in K [6]. The chestnuts from this region are famous for their aroma, sweetness, softness and glutinousness, but most notably their aroma, which is favored by consumers. The trademark of Wangmo chestnut ’Doujili’ was listed by the Hong Kong International Intellectual Property Trading Center in 2019 [7]. Thus, this industry has tremendous development potential. To date, researchers have studied only the fruit traits and quality of Chinese Yanshan [8], the main cultivar in the middle and lower reaches of the Yangtze River [9], and Beijing chestnut [1]. However, systematic research on Wangmo *C*. *mollissima* is lacking, and related research reports are very limited. To date, there have been no scientific reports on the fruit traits and quality of Wangmo *C*. *mollissima*, and no suitable cultivars have been bred, which seriously restricts the efficient development of this industry. Thus, screening of superior Wangmo *C*. *mollissima* plants was carried out, and excellent varieties or germplasms of *C*. *mollissima* cultivated in the Wangmo area were identified through comprehensive evaluation of their traits; the results could promote the diversification of Wangmo *C*. *mollissima* cultivars and the sustainable development of the industry.

Numerous comprehensive evaluation methods, such as factor analysis [10], the gray correlation method [11], the membership function method [12] and principal component analysis (PCA) [13], have been used to analyze *C*. *mollissima*. Despite various merits, factor analysis, gray correlation analysis and membership function analysis all show disadvantages when used for evaluating *C*. *mollissima* fruits, for which many traits need to be analyzed. Specifically, all these methods require the artificial assignment of weights to various traits or the artificial creation of ideal values and therefore exhibit subjectivity and limitations. In contrast to these methods, PCA can transform multiple observation indices into a few independent new indices, without artificially assigning weights to traits or creating ideal values, to simplify the data structure. In addition, PCA uses weighted summation to calculate the comprehensive score with all information completely and objectively accounted for, showing a relatively high level of objectivity [8]. PCA has been used to evaluate economic crops such as corn [14], pepper [15], grape [16], mango [17], tomato [18], and pomegranate [19].

In this study, thirty-two superior Wangmo *C*. *mollissima* plants were used as materials. By comparing their fruit phenotype and quality traits, they were divided into several major groups, and their phenotypic and quality traits were comprehensively evaluated to identify superior plants with better comprehensive quality. The results can provide a scientific basis for the effective development and utilization of chestnut resources.

## Materials and methods

### General information on the test site

The experimental site is located in Wangmo County, Guizhou Province (105°79’-106°05” E, 24°71’-24°94” N). This region is characterized by a mid-low mountainous valley terrain and the warm and humid climate of the South Asian subtropical region. It has sufficient light, abundant heat, and rain and heat in the same season. The average annual temperature is approximately 19°C, the average annual precipitation is 1222.5 mm, the frost-free period is 339 days, the extreme minimum temperature is -4.8°C, and the extreme maximum temperature is 41.8°C.

### Test materials

The samples involved in this study were sourced from four townships, i.e., Nagong, Nasha, Daguan, and Zhexiang, where Wangmo *C*. *mollissima* abounds. Based on the selection criteria of excellent tree growth, large single fruits, no pests or diseases, normal flowering followed by normal fruiting, and a stable yield, we performed observations for five consecutive years. Finally, the research group selected 32 representative superior Wangmo *C*. *mollissima* plants from the four representative towns as the breeding objects, among which 2 were from Daguan (labeled GM1 and GM2), 2 were from Nagong (GM3 and GM4), 23 were from Nasha (GM5-27) and 5 were from Zhexiang (GM28-32).

**Fig 1:**
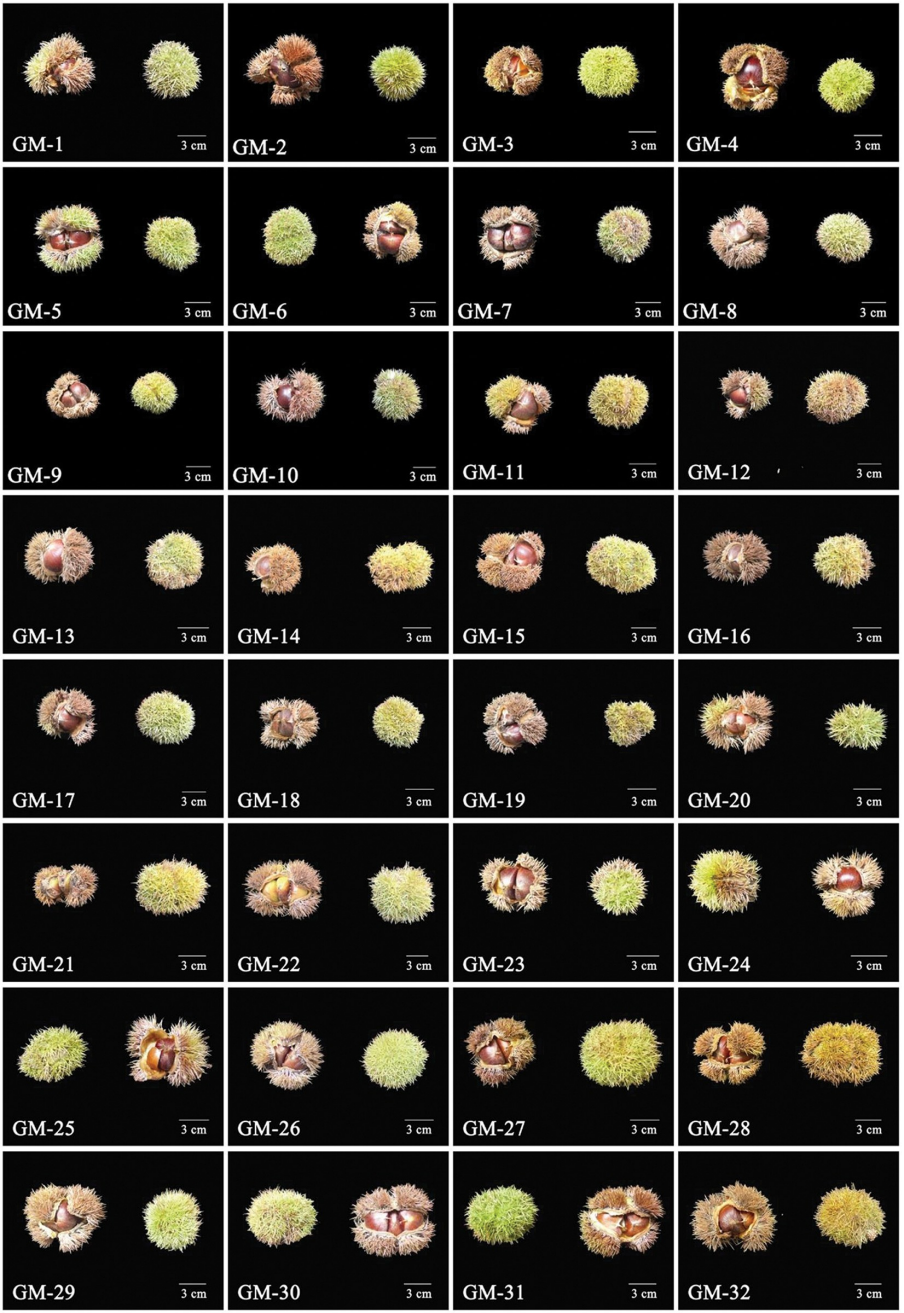
Morphological characteristics of mature chestnut bracts of 32 Wangmo *Castanea mollissima* plants.

**Fig 2:**
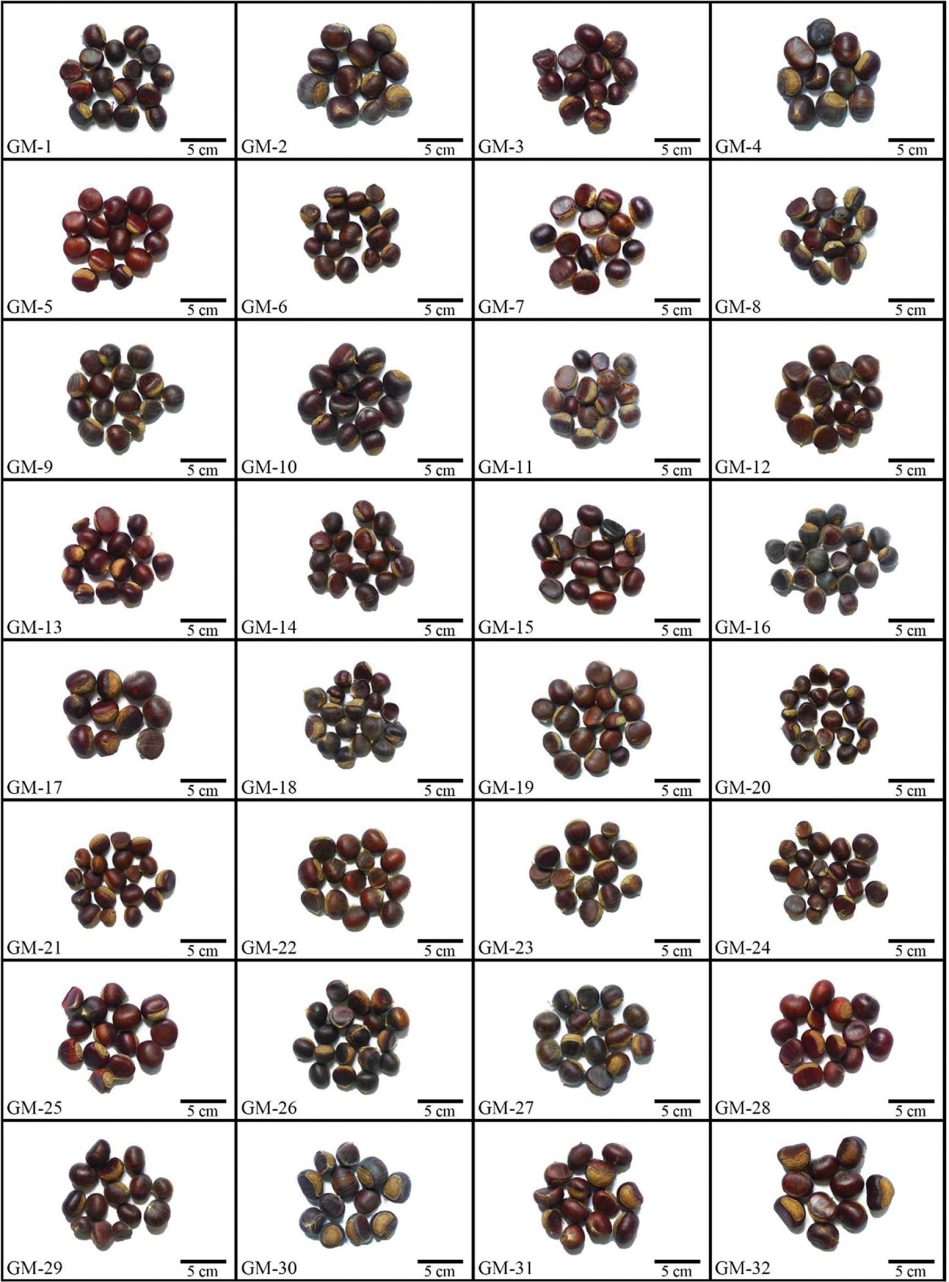
Morphological characteristics of the nuts of 32 *Castanea mollissima* plants.

The morphological characteristics of the mature chestnut bracts and nuts of the 32 Wangmo *C*. *mollissima* plants are shown in Figs 1 and 2. In late September 2021, when the *C*. *mollissima* fruits were fully mature and the chestnut bracts had naturally cracked, 50 mature fruits were randomly picked from the periphery of the tree for each superior plant. After bagging and labeling, samples were brought back to the laboratory to determine their fruit phenotype and quality.

### Test methods

#### Determination of fruit phenotypic traits

The transverse diameter, longitudinal diameter, shell thickness, transverse diameter, longitudinal diameter and thickness of nuts were measured by Vernier calipers, and the nut shape index was calculated. Fruit shape index = nut transverse diameter/nut longitudinal diameter, where the longitudinal diameter of the chestnut bract was measured by a steel ruler and a precision 1/100 balance was used to measure the total weight of a single chestnut bur. The measured data were the average of 30 chestnut bracts. Finally, the nuts were removed from the chestnut bracts, 30 nuts were randomly selected from each superior plant, and the weight of the nuts was determined. After the measurements had been recorded, the nuts were put into nylon mesh bags and dried in an oven. After drying, the dry weight of the nuts was measured, and the moisture content of the nuts was recorded; this process was repeated three times. The moisture content of each nut was calculated as follows [20]:

Nut moisture content (%) = (nut fresh weight-nut dry weight)/nut fresh weight × 100%

#### Determination of fruit quality traits

(1) Ash determination: A total of 1 g of sample was weighed and evenly spread in an ash dish. The ash dish was placed in the constant-temperature zone of a muffle furnace not exceeding 100°C, and the temperature in the furnace was slowly increased to 500°C and maintained for 30 min. Then, the sample was heated until the temperature reached 810°C and held at that temperature for 1 h. The ash dish was then removed and weighed after cooling to room temperature. Burning was performed every 20 min until the mass change was less than 0.001 g after two consecutive burnings, which was based on the mass after the last burning. The calculation of ash content in the sample was as follows [21]:

$$A=\frac{m1}{m2}\times \frac{100}{100-M}$$

where *A* is the ash content in the sample (g/100 g), *m1* is the mass of the remaining material after burning (g), *m2* is the weight of the sample (g), and *M* is the moisture content of the sample (%).

(2) Soluble sugar and starch were determined by the anthrone colorimetric method. The soluble protein was determined using Coomassie brilliant blue G-250 staining.

(3) Determination of fat: The nuts were dried in an oven at 40°C to a constant weight, and the chestnut kernels were removed and ground with a mortar. A 10 g sample was weighed and wrapped in a dried filter paper bag, and petroleum ether was used as an extractant. The fat content was determined by the Soxhlet extraction method and calculated according to the following formula [22]:

$$Fat\;content(\%)=\frac{Extracted\;fat\;mass}{Sample+Filter\;paper\;package\;mass}\times 100\%$$

(4) Determination of tannins: A total of 100 mL of chestnut extract was placed in a 500 mL beaker, water was added to 300 mL, and the mixture was preheated in a constant-temperature water bath at 35°C. A total of 20.00 mL of 1 mol/L zinc acetate solution was added to a 500 mL volumetric flask with 14 mL of ammonia water; the flask was shaken well, the white precipitate was dissolved with stirring, and the mixture was reacted for 30 min at a constant volume. A certain volume of reaction solution was pumped with a vacuum pump to obtain the filtrate. Then, 10.00 mL of the filtrate was added to 150 mL of deionized water with 12 mL of LNH_3_-NH_4_ Cl buffer solution (pH = 10) and 2–3 drops of chrome black T indicator. The solution was titrated with 0.05 mol/L EDTA until the color changed from wine red to dark blue, and blank correction was applied. The tannin content was calculated as follows [23]:

$$\mathrm{Tannin}\;\mathrm{content}\;(\%)=[(20\times C_{\mathrm{Zn}}‐50\times C_{\mathrm{EDTA}}\cdot V)\times 0.1556]\times 5\times 100/G(100‐X)$$

where 0.1556 is the proportional constant obtained in the experiment, *C*_*Zn*_ is the concentration of zinc acetate standard solution (mol/L), *c*_*EDTA*_ is the concentration of EDTA·2Na standard solution (mol/L), *V* is the corrected titration consumption of EDTA·2Na standard solution (mL), *G* is the mass of the chestnut sample (g), and *X* is the moisture content of the chestnut sample (%).

(5) Determination of total acid: A 20 g chestnut sample (accurate to 0.01 g) was placed in a mortar, and the same amount of carbon dioxide-free water was added to the sample. The sample was crushed in the mortar, mixed into a slurry and placed in a closed glass container. The total acid in the glass container was directly titrated with a standard solution by potentiometric titration. The total acid content was calculated according to the following formula [24]:

X=2×c×V

where *X* is the total acid content (g/kg) of the sample, *c* is the concentration of sodium hydroxide standard titration solution (mol/L), *V* is the volume of the standard titration solution used to consume sodium hydroxide (mL), and *2* is the coefficient for conversion to a 100 mL sample.

(6) Determination of Ca, K, Mg, Mn, Fe, and Zn:

First, an elemental stock solution was prepared, and an appropriate amount of single-element standard stock solution was diluted with nitric acid solution (5+95) to form a series of mixed standard working solutions. A solid sample of 0.2 g (accurate to 0.001 g) was weighed. The sample containing ethanol or carbon dioxide was first heated at low temperature on an electric heating plate to remove ethanol or carbon dioxide. Then, the sample was added to 5 mL of nitric acid, covered and placed for 1 h or overnight, and the lid was tightened. The digestion was carried out according to the standard operating steps of the microwave digestion instrument. After cooling, the digestion tank was removed, and the lid was slowly opened to release pressure. The inner lid was washed with a small amount of water. The digestion tank was placed on a temperature-controlled electric heating plate or in an ultrasonic water bath, heated at 100°C for 30 min, transferred to a 50 mL volumetric flask with water at a constant volume and shaken well. Blank test solution was prepared at the same time. The mixed standard solution was injected into an inductively coupled plasma mass spectrometer to determine the signal response values of the elements to be measured and the internal standard elements. The concentration of the elements to be measured was taken as the abscissa, and the ratio of the response signal values of the elements to be measured to those of the selected internal standard elements was taken as the ordinate; then, the standard curve was drawn. Blank solution and sample solution were injected into the inductively coupled plasma mass spectrometer, and the signal response values of the elements to be measured and the internal standard elements were determined. According to the standard curve, the concentrations of the elements to be measured in the digestion solution were determined. The contents of the high-content elements to be tested in the samples were calculated as follows [25]:

$$X=\frac{(P1-P2)\times V\times f}{m}$$

where *X* is the content of the element to be tested in the sample (mg/kg), *p1* is the mass concentration of the measured element in the sample solution (mg/L), *p2* is the mass concentration of the measured element in the sample blank solution (mg/L), *V* is the constant volume (mL) of the sample digestate, *f* is the dilution multiple of the sample, and m is the sample mass (g).

### Data management and analysis

All indicators were measured in triplicate. Excel 2019 was used to manage and calculate the mean values of relevant data. SPSS 20.0 was used to analyze the data. Single-factor analysis of variance (ANOVA) was used to analyze the excellent strains among groups. P<0.01 indicated a very significant difference, P<0.05 indicated a significant difference, and P>0.05 indicated that the difference was not significant.

SPSS 20.0 was used for PCA of the data. First, the original data were standardized, and then the eigenvalues, variance contribution rate, cumulative contribution rate and eigenvectors were calculated. Then, the principal component scores of each tested superior plant were calculated, and the variance contribution rate was used as the weight to obtain the comprehensive score of each parameter for the superior plant. Correlation analysis was also performed, and the coefficient of variation (CV) was obtained, with a value between -1 and 1. A CV value between 0.3 and 0.6 indicated a significant positive correlation, and a value over 0.6 indicated a very significant positive correlation. Finally, Origin was used for cluster analysis and correlation analysis, and Photoshop 2019 was used to process the images.

## Results and analysis

### Fruit phenotypic and quality characteristics analysis of different superior plants of *C*. *mollissima*

The morphological characteristics of chestnut bracts and nuts of 32 superior plants of Wangmo *C*. *mollissima* are shown in Figs 1 and 2. The fruit of Wangmo *C*. *mollissima* is spherical or oval, the chestnut bract is cyan, and the surface is prickly. The nut of Wangmo *C*. *mollissima* is large, the appearance is reddish brown or dark brown, and the surface is neat, full and bright. Different degrees of variation were observed in the 23 phenotypic (10 indices, designated X1-X10) and quality (13 indices, designated X11-X23) traits among the 32 superior plants of Wangmo *C*. *mollissima* (Tables 1 and 2). The CV of the 23 fruit traits ranged from 7.61% to 48.86%. The average CV of the 10 fruit phenotypic traits was 16.01%, and that of the 13 quality traits was 20.32%. In general, a CV greater than 15% indicates a high degree of variation in fruit traits. The CVs of 15 traits were higher than 15%, most of which had a coefficient between 10% and 20%. Among the considered traits of the 32 superior plants of Wangmo *C*. *mollissima*, the largest variation was observed in Mn content (48.86%), with a range of 271, followed by soluble sugar content (30.19%) and weight of a single chestnut bract (27.46%), with ranges of 13.07 and 29.66, respectively. These three traits have high improvement potential and rich heritability. The smallest CVs were observed for starch (7.61%), fruit shape index (9.10%), and longitudinal diameter (10.25%), and the ranges were 18.76, 0.29, and 12.22, respectively. In summary, *C*. *mollissima* is stable in terms of starch content, fruit shape index and longitudinal diameter, and the improvement potential is small. In terms of fruit phenotypic traits, the best fruits were observed on the GM-32 plant. The chestnut bracts were large with short bract thorns, and the nuts were large with a low moisture content. In terms of fruit quality traits, the best plant was also GM-32, which had the highest levels of Fe, Mg and Zn.

**Table 1:** Statistical results of the fruit phenotypic characteristics of each superior Wangmo *C*. *mollissima* plant.

Plant no.	X1 (weight of a single chestnut bract/g)	X2 (diameter of the chestnut bract/mm)	X3 (longitudinal diameter of the chestnut bract/mm)	X4 (thickness of the chestnut shell/mm)	X5 (longitudinal diameter of the bract thorn/mm)	X6 (nut diameter/mm)	X7 (longitudinal diameter of the nut/mm)	X8 (thickness of the nut/mm)	X9 (fruit shape index)	X10 (moisture content of the nut/%)
GM-1	32.55±1.94^bcdefg^	45.96±2.82^defghi^	45.30±2.43^bcd^	2.90±0.59^a^	0.90±0.10^cdefgh^	25.16±0.31^hijkl^	22.57±0.87^efghij^	19.22±2.31^bcdefghij^	0.90±0.05^cdefghij^	57.4±0.92 ^l^
GM-2	20.42±3.29^efgh^	43.98±8.30^defghi^	33.64±5.95^jkl^	2.17±0.52^abcd^	0.85±0.21^efghij^	31.74±1.44^abc^	25.53±0.93^abcd^	22.11±1.99^abcdef^	0.81±0.02^hijkl^	86.61±0.88^a^
GM-3	18.05±3.58^gh^	40.17±5.75^hij^	37.48±3.92^fghijk^	2.48±0.70^abc^	0.72±0.20^ghijkl^	32.10±3.42^abc^	24.85±0.90^bcde^	23.41±1.05^ab^	0.78±0.08^jkl^	59.94±1.21^k^
GM-4	23.22±3.14^efgh^	48.58±5.11^cdefgh^	44.84±4.70^bcde^	1.84±0.48^bcd^	0.79±0.07^efghijk^	29.44±3.46^cdef^	23.04±1.42^defghi^	22.28±1.44^abcdef^	0.79±0.08^ijkl^	72.09±1.32^d^
GM-5	34.78±4.91^bcde^	47.61±6.02^cdefgh^	43.47±3.31^cdefg^	1.65±0.19^cd^	0.8±0.13^efghijk^	26.46±2.57^fghij^	24.72±1.13^bcdef^	18.17±4.32^fghijk^	0.94±0.09^abcdef^	71.76±0.94^d^
GM-6	22.13±2.61^efgh^	42.25±4.25^fghij^	34.49±2.65^jkl^	2.19±0.67^abcd^	0.54±0.15^l^	21.72±3.21^l^	20.29±2.00^jkl^	17.19±2.20^ghijkl^	0.94±0.09^abcdef^	65.23±0.75^hi^
GM-7	33.53±5.68^bcdef^	51.96±5.56^cdef^	44.35±3.11^cdef^	2.11±0.5^2abcd^	0.84±0.13^efghij^	25.25±2.39^hijkl^	22.24±2.94^efghij^	18.5±2.97^defghijk^	0.88±0.07cdefghijk	69.96±0.68^e^
GM-8	32.07±4.49bcdefg	56.64±6.00bc	50.15±4.31^abc^	2.28±0.56^abcd^	0.78±0.20^fghijk^	30.53±1.83bcde	26.45±1.12^ab^	22.85±0.85^abcd^	0.87±0.03^cdefghijkl^	63.51±0.71^ij^
GM-9	23.76±6.10^efgh^	43.77±5.91^defghi^	33.19±3.59^kl^	1.95±0.30^abcd^	0.64±0.11^jkl^	25.00±1.70^hijkl^	24.01±0.64^bcdefgh^	19.16±2.24^bcdefghij^	0.96±0.05^abcd^	66.75±0.82^gh^
GM-10	29.42±3.61^bcdefg^	52.56±6.47^cde^	45.49±5.99^bcd^	2.12±0.69^abcd^	0.97±0.12^cdef^	28.14±2.88^defgh^	23.49±2.73^cdefghi^	20.98±5.70^abcdefg^	0.83±0.08^efghijkl^	67.59±0.36^fg^
GM-11	24.42±3.18^defgh^	43.17±4.32^efghi^	35.98±3.57^ijkl^	1.30±0.30^d^	0.65±0.06^ijkl^	27.38±1.57^defghi^	24.08±1.55^bcdefg^	22.59±1.74^abcde^	0.88±0.03^cdefghijk^	65.01±0.68^hi^
GM-12	29.84±3.52^bcdefg^	44.82±8.33^defghi^	40.32±2.98^defghij^	2.42±0.88^abc^	0.79±0.10^efghijk^	26.64±3.51^fghij^	25.37±2.35^bcd^	17.54±0.93^ghijk^	0.96±0.05^abcd^	68.81±1.23^ef^
GM-13	17.62±3.67^gh^	41.54±4.13^hij^	36.36±3.70^hijk^	2.26±0.62^abcd^	0.60±0.06^kl^	24.5±0.51^hijkl^	22.35±0.47^efghij^	19.01±2.85^cdefghij^	0.91±0.04^bcdefgh^	68±1.12^fg^
GM-14	27.95±4.28^cdefgh^	42.99±4.55^efghi^	33.56±4.19^jkl^	1.59±0.45^cd^	1.01±0.17^cde^	24.19±1.81^ijkl^	23.03±2.56^defghi^	15.19±2.96^jkl^	0.95±0.05^abcde^	65.08±1.23^hi^
GM-15	41.42±6.57^bc^	56.58±2.57^bc^	43.17±4.74^defgh^	2.00±0.37^abcd^	0.63±0.08^jkl^	27.08±1.51^efghi^	21.33±0.60^ijkl^	17.08±2.35^ghijkl^	0.79±0.03^ijkl^	62.93±1.25^j^
GM-16	28.95±3.32^cdefgh^	46.77±3.02^defgh^	43.46±3.56cdefg	1.57±0.39^cd^	0.70±0.08^hijkl^	22.64±0.78^kl^	21.90±1.29^ghijk^	15.76±0.16^ijkl^	0.97±0.04^abc^	66.69±0.89^gh^
GM-17	34.30±5.21^bcde^	49.95±6.10^cdefgh^	46.76±8.43^abcd^	2.28±0.40^abcd^	1.08±0.21^bcd^	30.82±2.37^bcd^	23.06±0.74^defghi^	23.17±0.95^abc^	0.75±0.05^l^	77.35±0.92^b^
GM-18	14.30±1.31^h^	36.91±2.93^ij^	33.22±4.05^kl^	2.31±0.59^abcd^	1.08±0.16^bcd^	30.52±3.35^bcde^	23.62±1.19^cdefghi^	22.47±1.34^abcdef^	0.78±0.09^jkl^	70.07±0.98^e^
GM-19	26.04±3.98^defgh^	49.07±7.82^cdefgh^	41.49±6.11^defghi^	2.18±0.30^abcd^	0.81±0.20^efghijk^	21.62±2.67 ^l^	21.92±1.67^ghijk^	17.21±2.35^ghijkl^	1.04±0.22^a^	75.35±0.87^c^
GM-20	24.59±2.52^defgh^	52.25±7.75^cde^	32.17±5.21^kl^	1.75±0.84^bcd^	0.91±0.10^cdefgh^	17.15±2.63^m^	15.71±2.55^m^	12.94±2.61^l^	0.92±0.11^abcdefgh^	53.27±0.92^n^
GM-21	18.79±1.42^fgh^	33.56±6.28^j^	29.38±3.16^l^	1.91±0.41^abcd^	0.89±0.33^cdefgh^	23.04±1.10^jkl^	21.37±0.54^hijkl^	14.23±3.46^kl^	0.93±0.03^abcdefg^	63.59±1.32^ij^
GM-22	42.47±6.94^bc^	51.59±4.77^cdefg^	52.67±6.74^a^	1.90±0.5^abcd^	1.01±0.20cde	25.02±2.40^hijkl^	25.28±1.32bcd	17.47±1.65ghijk	1.02±0.15^ab^	62.95±1.62^j^
GM-23	18.04±2.33^gh^	41.86±6.01^ghij^	38.14±1.46^efghijk^	1.83±0.52^bcd^	0.87±0.04^defghi^	24.82±1.22^hijkl^	22.04±0.89^ghijk^	18.49±1.53^defghijk^	0.89±0.02^cdefghij^	75.7±0.98^c^
GM-24	19.60±2.76^efgh^	44.18±5.99^defghi^	38.21±3.61^efghijk^	1.90±0.49abcd	0.75±0.15^fghijkl^	23.20±2.39^jkl^	19.55±2.34^kl^	15.75±3.25ijkl	0.84±0.02^defghijkl^	62.66±2.36^j^
GM-25	34.06±3.77^bcde^	45.74±3.59^defghi^	45.62±4.99^bcd^	2.45±0.61^abc^	1.23±0.22^b^	24.33±1.87^ijkl^	23.75±1.76^cdefghi^	16.21±3.13^hijkl^	0.98±0.10^abc^	60.81±2.43^k^
GM-26	34.42±3.35^bcde^	53.39±7.28^cd^	45.62±3.43bcd	1.48±0.55^cd^	0.87±0.10^defghi^	23.58±1.39^ijkl^	19.41±3.46 ^l^	15.34±2.27^jkl^	0.82±0.12^fghijkl^	64.05±1.89^ij^
GM-27	38.88±4.06^bcd^	49.59±7.85^cdefgh^	44.03±4.1^cdef^	2.73±0.74^ab^	0.75±0.12^fghijkl^	24.71±3.26^hijkl^	22.15±1.48^fghij^	16.62±1.76^ghijkl^	0.90±0.10^bcdefghi^	67.58±1.95^fg^
GM-28	38.96±3.30^bcd^	48.42±7.53^cdefgh^	36.98±3.83^ghijk^	1.72±0.48bcd	0.93±0.12^cdefg^	25.72±2.87^ghijk^	22.61±2.09^efghij^	18.31±5.31^efghijk^	0.88±0.08^cdefghijk^	55.49±0.65^m^
GM-29	43.96±4.84^b^	62.32±8.53b	51.59±5.22^ab^	2.34±0.37abc	0.96±0.12^cdef^	28.99±3.07^cdefg^	25.73±2.39abc	20.09±4.97abcdefghi	0.89±0.03^cdefghij^	57.97±0.98 ^l^
GM-30	23.73±2.78^efgh^	42.29±6.75^fghij^	35.99±3.37ijkl	1.89±0.50^abcd^	1.50±0.04^a^	31.88±3.79^abc^	25.99±1.69^abc^	21.90±3.50abcdef	0.82±0.05^fghijkl^	57.96±1.65^l^
GM-31	34.18±3.48^bcde^	63.82±8.25^b^	41.77±9.33^defghi^	2.25±0.99^abcd^	0.85±0.15^efghij^	33.11±2.62^ab^	25.28±0.54^bcd^	24.32±1.54^a^	0.77±0.05^kl^	55.32±1.32 ^m^
GM-32	32.47±3.65^a^	78.73±2.95^a^	52.56±3.49^a^	1.68±0.20^cd^	1.10±0.08^bc^	34.69±3.89^a^	27.93±1.65^a^	20.62±5.58^abcdefgh^	0.81±0.06^ghijkl^	57.43±0.87^l^
Minimum	14.30	33.56	29.38	1.30	0.54	17.15	15.71	12.94	0.75	53.27
Maximum	43.96	78.73	52.67	2.90	1.50	34.69	27.93	24.32	1.04	86.61
Range	29.66	45.17	23.29	1.60	0.96	17.54	12.22	11.38	0.29	33.34
Mean	28.72	48.53	40.98	2.04	0.87	26.6	23.15	18.94	0.88	65.47
Standard deviation	7.89	8.48	6.22	0.36	0.19	3.86	2.37	2.96	0.08	7.12
Coefficient of variation (%)	27.46	17.47	15.17	17.48	22.14	14.51	10.25	15.61	9.10	10.87

Notes: Data are presented as the mean ± standard deviation; different letters in the same column indicate a significant difference (P<0.05), according to *t* tests.

**Table 2:** Statistical results of fruit quality traits for each superior Wangmo *C*. *mollissima* plant.

Plant no.	X11 (calcium, mg/kg)	X12 (potassium, mg/kg)	X13 (magnesium, mg/kg)	X14 (manganese, mg/kg)	X15 (iron, mg/kg)	X16 (zinc, mg/kg)	X17 (ash content, g/100 g)	X18 (soluble protein, %)	X19 (tannin, mg/kg)	X20 (soluble sugar, %)	X21 (starch, %)	X22 (fat, g/100 g)	X23 (total acid, g/kg)
GM-1	830	8602	1079	305.00	20.10	13.10	1.59	0.40	3700	12.34	56.13	1.70	1.84
GM-2	436	8892	1059	86.35	15.32	10.26	1.95	0.43	3058	15.82	50.62	1.60	1.94
GM-3	422	6640	918	65.90	21.80	10.90	1.15	0.37	3697	18.10	44.09	2.40	1.37
GM-4	356	6304	904	53.70	19.60	10.70	1.03	0.27	2656	6.00	54.65	1.70	1.04
GM-5	409	5748	1067	72.00	19.10	13.50	1.17	0.38	2973	8.73	58.07	1.60	2.24
GM-6	512	7225	1153	153.00	15.10	11.00	1.52	0.34	3169	9.91	53.37	1.60	1.73
GM-7	590	6570	1187	129.00	16.00	10.60	1.11	0.37	3353	10.55	54.58	1.90	1.37
GM-8	594	9271	962	72.40	15.40	8.47	1.40	0.33	3299	13.16	50.17	2.20	1.66
GM-9	518	6497	1043	73.50	17.00	9.77	1.08	0.31	3392	10.63	57.58	1.80	1.77
GM-10	590	8225	980	201.00	18.90	14.30	1.54	0.30	3717	8.52	55.12	2.60	1.44
GM-11	480	7631	1098	134.00	16.60	11.10	1.39	0.39	3268	11.80	54.66	2.10	1.24
GM-12	510	7482	891	188.00	16.20	10.60	1.16	0.29	3099	9.41	58.40	1.90	1.66
GM-13	628	7174	1169	97.70	15.20	10.90	1.22	0.39	3372	13.84	54.67	1.50	1.91
GM-14	503	7323	1199	122.00	15.20	12.90	1.56	0.40	2882	11.12	57.42	1.90	1.53
GM-15	448	6834	1018	160.00	18.50	11.40	1.38	0.39	2920	9.40	56.49	1.10	1.63
GM-16	459	4948	817	34.00	14.20	9.83	1.28	0.29	2917	9.70	57.99	2.30	1.44
GM-17	435	5734	1003	56.20	18.10	15.90	1.56	0.30	3522	5.03	59.62	1.90	2.00
GM-18	566	7256	1141	109.00	18.10	11.20	1.98	0.32	3143	11.05	53.54	1.80	1.57
GM-19	675	7683	1582	143.00	28.70	19.70	2.26	0.21	3838	5.77	51.09	0.30	1.53
GM-20	606	7830	1278	186.00	14.50	12.30	2.04	0.30	2937	11.59	52.00	2.30	2.17
GM-21	566	7704	1178	106.00	22.50	13.20	2.17	0.37	2904	8.37	52.61	1.80	2.12
GM-22	491	8611	969	75.30	20.90	10.10	2.16	0.41	2972	15.59	49.50	2.10	1.92
GM-23	457	9083	1187	98.50	18.80	10.80	2.10	0.32	3459	6.46	58.23	1.00	1.44
GM-24	543	8370	932	224.00	19.10	11.90	2.08	0.35	4634	12.27	51.54	1.50	1.50
GM-25	582	8465	1253	121.00	19.30	11.40	2.13	0.37	3217	7.96	58.23	1.30	1.71
GM-26	679	7795	1127	151.00	16.90	12.60	1.96	0.45	2990	11.51	62.85	1.90	1.77
GM-27	489	8523	1120	93.50	16.90	12.60	1.96	0.45	2990	11.51	62.85	1.90	1.77
GM-28	821	9081	1336	138.00	21.20	11.20	2.07	0.47	3941	9.91	52.59	2.00	2.10
GM-29	401	8820	1045	85.20	16.50	11.30	1.89	0.43	3143	13.79	51.31	1.70	1.92
GM-30	648	7552	1288	74.00	20.10	11.40	1.62	0.38	3408	15.03	52.90	1.90	1.50
GM-31	542	8323	1225	48.60	16.70	9.96	1.89	0.57	3437	13.60	47.66	1.60	1.83
GM-32	580	8654	908	93.00	15.90	8.22	1.28	0.44	3317	17.84	44.66	2.20	1.86
Minimum	356	4948	817	34.00	14.20	8.22	1.03	0.21	2656	5.03	44.09	0.30	1.04
Maximum	830	9271	1582	305.00	28.70	19.70	2.26	0.57	4634	18.1	62.85	2.60	2.24
Range	474	4323	765	271.00	14.50	11.48	1.23	0.36	1978	13.07	18.76	2.30	1.20
Mean	543	7652	1097	117.20	18.13	11.64	1.65	0.37	3310	10.99	53.93	1.77	1.70
SD	109	1069	155	57.30	2.90	2.11	0.39	0.68	388	3.32	4.11	0.44	0.28
CV, %	20	13.97	14.15	48.86	16.00	18.17	23.52	18.72	11.73	30.19	7.61	24.80	16.38

Notes: SD, standard deviation; CV, coefficient of variation.

### Correlation analysis of fruit phenotypic and quality traits

**Fig 3:**
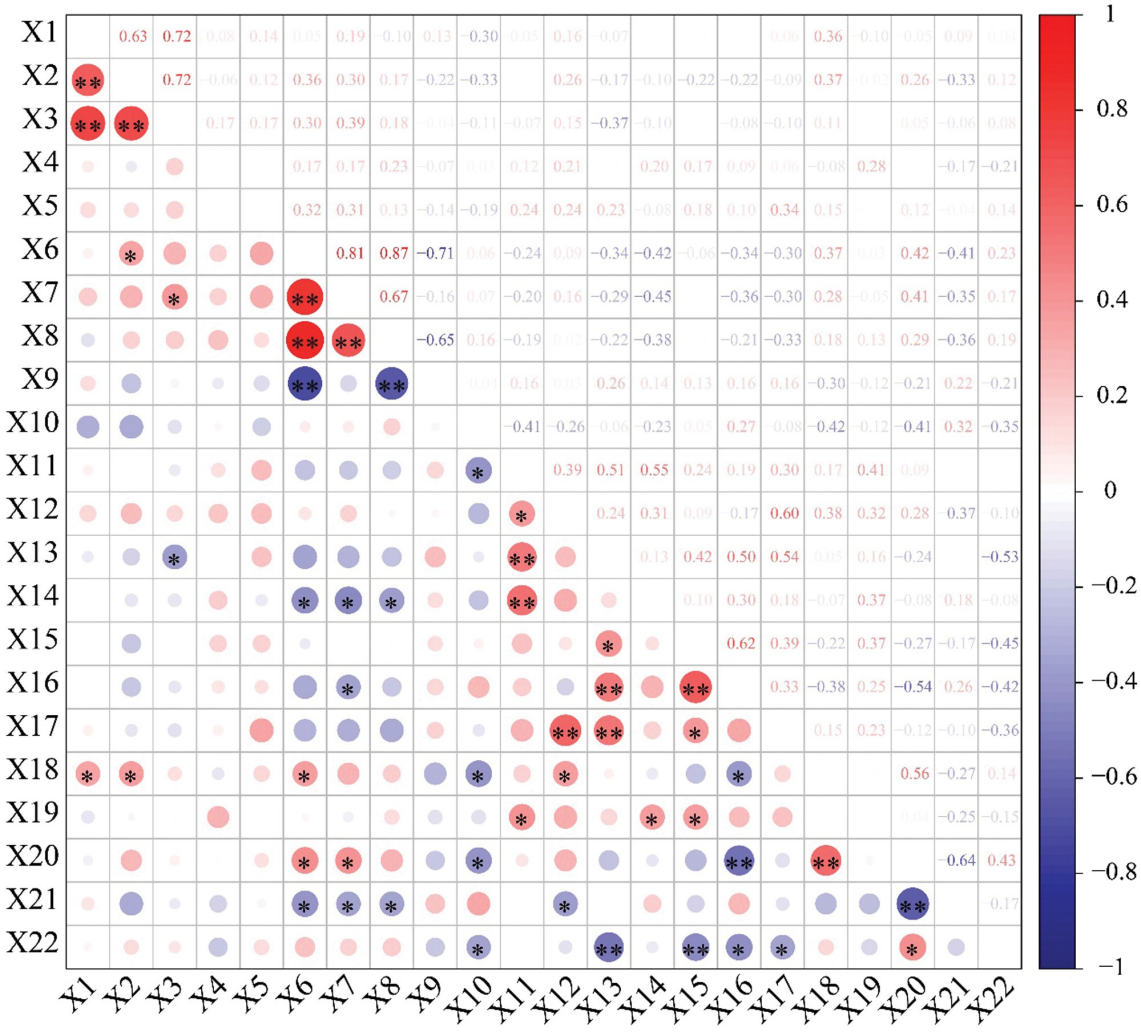
Correlation matrix of 23 fruit trait indices. Note: X1-X23 represent the weight of a single chestnut bract, diameter of the chestnut bract, longitudinal diameter of the chestnut bract, thickness of the chestnut shell, length of the bract thorn, nut diameter, longitudinal diameter of the nut, thickness of the nut, fruit shape index, moisture content of the nut, Ca, K, Mg, Mn, Fe, Zn, ash content, soluble protein, tannin, soluble sugar, starch, fat, and total acid, respectively.

Correlation analysis of 23 fruit trait indices of Wangmo *C*. *mollissima* was carried out, and the results showed different degrees of correlation among the indices (Fig 3). There were very significant positive correlations for the weight of a single chestnut bract with the transverse diameter and longitudinal diameter of chestnut bracts, and there was a significant positive correlation with soluble protein, with correlation coefficients of 0.63, 0.72 and 0.36, respectively. There were very significant positive correlations for the transverse diameter of chestnut bracts with the longitudinal diameter of chestnut bracts and significant positive correlations with the transverse diameter of nuts and soluble protein, with correlation coefficients of 0.72, 0.36 and 0.37, respectively. There was a significant positive correlation for the longitudinal diameter of chestnut bracts with that of nuts and a significant negative correlation with Mg, with correlation coefficients of 0.39 and -0.37, respectively. There were very significant positive correlations among nut transverse diameter, nut longitudinal diameter and nut thickness, with correlation coefficients of 0.81, 0.87 and 0.67, respectively. Nut transverse diameter and nut longitudinal diameter were both positively correlated with soluble sugar (correlation coefficients: 0.42 and 0.41, respectively), and they were all negatively correlated with Mn and starch (correlation coefficients: 0.42, -0.45, and -0.38 for Mn and -0.41, -0.35 and -0.36 for starch). Moreover, nut transverse diameter and nut thickness showed very significant correlations with fruit shape index, with correlation coefficients of 0.87 and 0.67, respectively. There was a significant positive correlation between nut transverse diameter and soluble protein (correlation coefficient, 0.37), and there was a significant negative correlation between nut longitudinal diameter and Zn (correlation coefficient, -0.376). The moisture content of nuts showed a significant correlation with the Ca, soluble protein, soluble sugar and fat contents, with correlation coefficients of -0.41, -0.42, -0.41 and -0.35, respectively. The correlations between mineral elements were notable and strong. The content of one component could be predicted according to that of another component. The ash content was only significantly negatively correlated with the fat content (correlation coefficient, -0.36). Soluble protein showed a very significant positive correlation with the soluble sugar content (0.56) and a significant positive correlation with the total acid content (0.36). There was a significant positive correlation of the soluble sugar content with the fat content (0.43) but a significant negative correlation with the starch content (-0.64). Although there were different degrees of correlation between other nutritional indicators, the correlations did not reach a significant level.

### Cluster analysis of the Wangmo *C*. *mollissima* system

**Fig 4:**
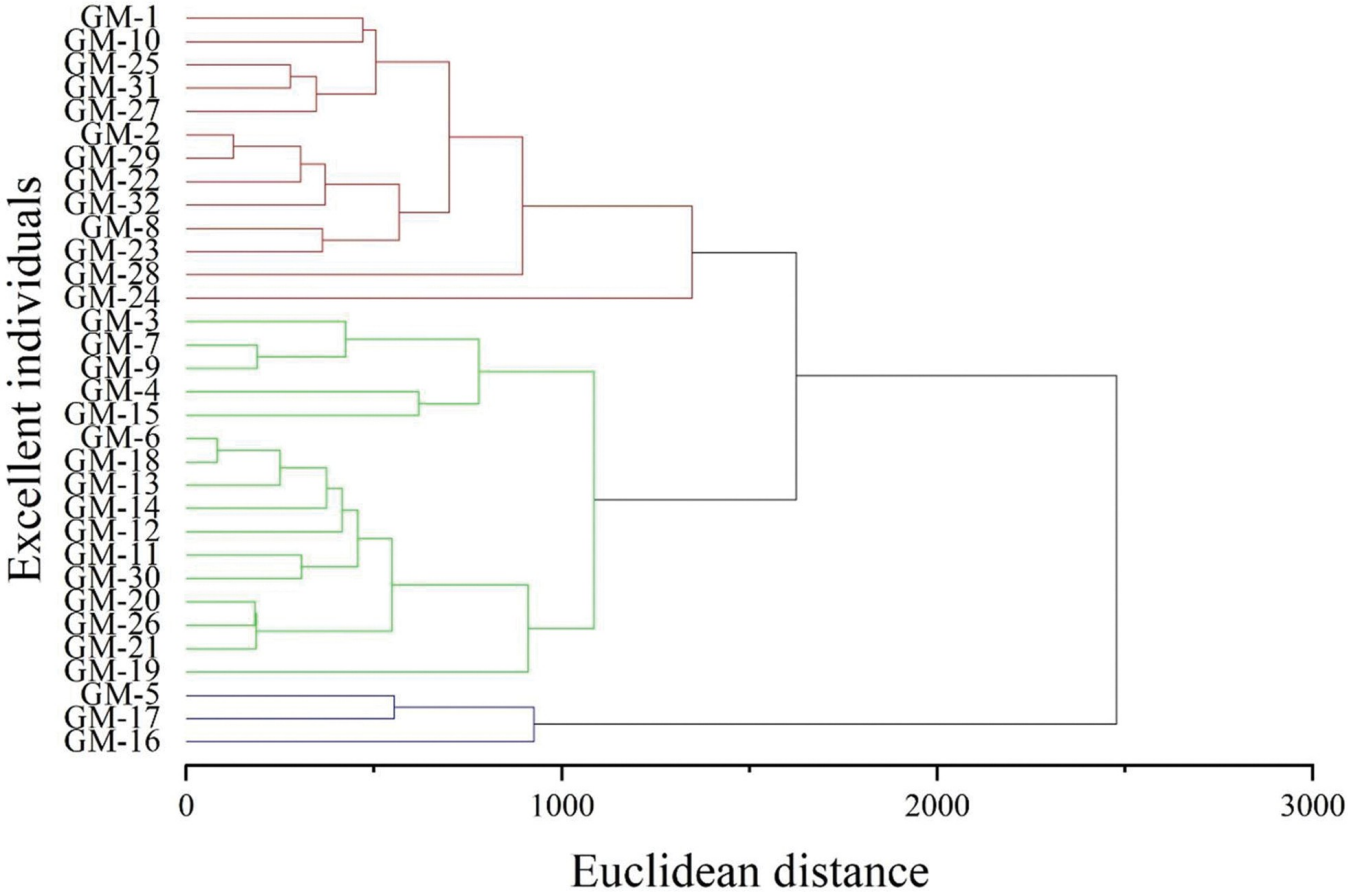
Cluster analysis of 32 superior Wangmo *C*. *mollissima* plants.

Cluster analysis was performed on the 32 superior Wangmo *C*. *mollissima* plants based on 23 fruit traits, and a tree diagram was constructed, as shown in Fig 4. At a Euclidean distance of 1500, the 32 superior plants could be clustered into three groups: Group I included 13 superior plants, namely, GM1, GM-2, GM-8, GM-10, GM-22, GM-23, GM-24, GM-25, GM-27, GM-28, GM-29, GM-31 and GM-32; Group II included 16 superior plants, namely, GM-3, GM-4, GM-6, GM-7, GM-9, GM-11, GM-12, GM-13, GM-14, GM-15, GM-18, GM-19, GM-20, GM-21, GM-26 and GM-30; and Group III included three asexual lines, namely, GM-5, GM-16 and GM-17.

Table 3 shows the average performance of the three clusters. There were significant differences in the longitudinal diameter of chestnuts and the Ca, K, Mn, ash, soluble sugar and starch contents among the three clusters. The first group of plants produced large fruits. The Ca, K, Mn, ash and soluble sugar contents were significantly higher than those in the other groups, but the starch content was significantly lower than that in the other groups. The second group included plants producing small fruits, and its Ca, K, Mn, ash, soluble sugar and starch contents were intermediate. The third group, including plants with medium-sized fruits, had significantly lower Ca, K, Mn, ash and soluble sugar contents than the other groups, but the starch content was significantly higher than that of the other groups.

**Table 3:** The main characteristics of different cluster groups of Wangmo *C*. *mollissima*.

Index	Cluster group 1	Cluster group 2	Cluster group 3
X1 (g)	32.08±8.40^a^	25.24±7.00^a^	32.68±3.24^a^
X2 (mm)	52.72±10.40^a^	45.21±6.27^a^	48.11±1.65^a^
X3 (mm)	44.32±6.31^a^	37.60±5.06^b^	44.56±1.90^a^
X4 (mm)	2.17±0.37^a^	1.98±0.33^a^	1.83±0.39^a^
X5 (mm)	0.92±0.14^a^	0.83±0.23^a^	0.86±0.20^a^
X6 (mm)	27.70±3.77^a^	25.69±4.03^a^	26.64±4.09^a^
X7 (mm)	24.03±2.26^a^	22.41±2.53^a^	23.23±1.42^a^
X8 (mm)	19.46±2.66^a^	18.50±3.26^a^	19.03±3.78^a^
X9	0.87±0.07^a^	0.88±0.08^a^	0.88±0.12^a^
X10 (%)	63.92±8.93a	65.51±5.48a	71.93±5.33a
X11 (mg/kg)	565.85±130.77^a^	544.19±92.47^ab^	434.33±25.01^b^
X12 (mg/kg)	8686.15±322.69^a^	7218.75±502.18^b^	5476.67±457.89^c^
X13 (mg/kg)	1081.15±135.07^a^	1135.88±170.76^a^	962.33±129.87^a^
X14 (mg/kg)	126.30±73.60^a^	121.61±41.48^a^	54.07±19.09^b^
X15 (mg/kg)	18.21±2.03^a^	18.25±3.68^a^	17.13±2.59^a^
X16 (mg/kg)	10.99±1.68^a^	11.89±2.28^a^	13.08±3.06^a^
X17 (g/100 g)	1.85±0.30^a^	1.54±0.42^ab^	1.34±0.20^b^
X18 (%)	3.98±0.75^a^	3.47±0.60^a^	3.24±0.47a
X19 (mg/kg)	3498.69±441.27^a^	3189.25±314.19^a^	3137.33±334.31^a^
X20 (%)	11.86±3.63^a^	10.88±3.07^ab^	7.82±2.46^b^
X21 (%)	52.25±3.63^b^	54.43±3.07^ab^	58.56±2.46^a^
X22 (g/100 g)	1.77±0.43^a^	1.74±0.49^a^	1.93±0.35^a^
X23 (g/kg)	1.75±0.22^a^	1.62±0.30^a^	1.89±0.41^a^

Notes: X1-X23 represent the weight of a single chestnut bract, diameter of the chestnut bract, longitudinal diameter of the chestnut bract, thickness of the chestnut shell, longitudinal diameter of the bract thorn, nut diameter, longitudinal diameter of the nut, thickness of the nut, fruit shape index, moisture content of the nut, calcium, potassium, magnesium, manganese, iron, zinc, ash content, soluble protein, tannin, soluble sugar, starch, fat, and total acid, respectively. Data are presented as the mean ± standard deviation. Different letters in the same row indicate a significant difference (P<0.05).

### Comprehensive evaluation of Wangmo *C*. *mollissima* fruit

To avoid the influence of different dimensions on the analysis results, SPSS 20.0 was used to standardize the original data of the 23 fruit phenotype and quality indicators, and then PCA was carried out to extract 7 common factors with eigenvalues greater than 1. Table 4 shows that the eigenvalues of the first seven principal components were greater than 1, i.e., 5.179, 3.658, 2.613, 2.160, 1.621, 1.230 and 1.046, and the cumulative contribution rate was 76.122%. The first principal component was a comprehensive index composed of X2, X6, X7, X8, X13, X16, X20, X21 and X22, and the variance contribution rate was 22.518%. The second principal component was a comprehensive index composed of X10, X11, X12, X17, X18 and X23, and the variance contribution rate was 15.906%. The third principal component was composed of X4 and X15, and the variance contribution rate was 11.361%. The fourth principal component was composed of X1 and X3, and the variance contribution rate was 9.391%. The fifth principal component was composed of X14 and X19, and the variance contribution rate was 7.049%. The sixth principal component mainly included X5, and the variance contribution rate was 5.348%. The seventh principal component included X9 as the main evaluation index, and the variance contribution rate was 4.549%. Based on these findings, these seven principal components could replace most of the information of the original 23 traits, so they were used to represent the original 23 indicators in a comprehensive quality evaluation of Wangmo *C*. *mollissima*.

**Table 4:** Component loading matrix of the traits.

Fruit index	Principal component						
	F1	F2	F3	F4	F5	F6	F7
X1	0.221	0.381	-0.365	0.733	0.076	0.019	0.055
X2	0.544	0.369	-0.255	0.462	0.122	-0.025	0.080
X3	0.438	0.215	-0.094	0.75	0.291	-0.022	-0.108
X4	0.037	0.231	0.412	0.107	0.394	-0.268	0.149
X5	0.164	0.420	0.183	0.135	-0.373	0.556	-0.238
X6	0.854	0.009	0.454	0.037	-0.086	0.150	0.102
X7	0.747	0.048	0.316	0.197	-0.153	-0.099	-0.228
X8	0.698	-0.099	0.586	-0.049	0.030	0.121	0.112
X9	-0.539	0.058	-0.365	0.177	-0.074	-0.373	-0.481
X10	-0.189	-0.563	0.449	0.216	-0.164	-0.160	0.270
X11	-0.294	0.677	-0.055	-0.216	0.202	0.321	-0.042
X12	0.099	0.758	0.066	-0.109	0.013	-0.280	0.123
X13	-0.563	0.464	0.230	-0.057	-0.397	0.060	0.031
X14	-0.445	0.373	-0.132	-0.179	0.599	0.157	0.175
X15	-0.381	0.316	0.600	0.195	-0.027	0.004	-0.368
X16	-0.656	0.109	0.391	0.314	0.002	0.267	-0.017
X17	-0.418	0.627	0.130	0.000	-0.346	-0.121	0.103
X18	0.488	0.508	-0.242	-0.141	-0.271	-0.008	0.372
X19	-0.127	0.434	0.434	-0.194	0.495	-0.005	0.011
X20	0.645	0.310	-0.159	-0.476	-0.038	-0.148	-0.151
X21	-0.485	-0.387	-0.244	0.321	0.006	0.382	0.308
X22	0.507	-0.107	-0.408	-0.298	0.155	0.395	-0.266
X23	-0.064	0.418	-0.345	0.058	-0.370	-0.051	0.221
Eigenvalue	5.193	3.660	2.620	2.172	1.623	1.215	1.050
Variance contribution rate\%	22.579	15.912	11.391	9.446	7.057	5.285	4.563
Cumulative contribution rate\%	22.579	38.491	49.882	59.328	66.385	71.669	76.233

Based on the proportions of the total contribution rate corresponding to the seven principal components, the comprehensive evaluation scoring model of the fruit traits for the 32 superior plants was as follows: Y = 0.296F1+0.209F2+0.149F3+0.123F4+0.093F5+0.070F6+0.060F7. The comprehensive scores of the 32 superior plants were calculated according to this model and used to sort the plants. The results are shown in Table 5. GM32 scored the highest for the first principal component, with a value of 2.552; GM28 had the highest score for the second principal component, 2.069. GM19 scored the highest for the third principal component, at 2.507; GM17 scored the highest for the fourth principal component, at 1.854. GM1 had the highest score in the fifth principal component, 2.449. GM30 scored the highest for the sixth principal component, at 2.249; GM2 had the highest score for the seventh principal component, at 2.037. In terms of comprehensive quality, the 32 Wangmo *C*. *mollissima* plants were ranked as follows: GM32>GM31>GM29>GM1>GM8>GM17>GM10>GM30>GM3>GM28>GM27>GM15>GM22>GM2>GM18>GM25>GM7>GM4>GM26>GM24>GM12>GM19>GM11>GM5>GM23>GM13>GM9>GM14>GM6>GM16>GM21>GM20.

**Table 5:** Thirty-two principal component scores and comprehensive scores of superior plants.

Superior plant number	Principal component score							Comprehensive score	Ranking
	F1	F2	F3	F4	F5	F6	F7		
GM1	-0.520	1.657	0.058	-0.159	2.449	0.693	0.541	0.490	4
GM 2	0.854	-0.312	1.023	-1.011	-1.614	-1.394	2.037	0.090	14
GM 3	1.397	-0.588	1.466	-1.776	0.951	-0.484	-1.522	0.254	9
GM 4	0.532	-2.182	0.849	0.781	0.271	0.395	-0.385	-0.046	18
GM 5	-0.129	-0.977	-0.492	1.395	-1.165	0.150	0.048	-0.239	24
GM 6	-0.807	-0.632	-0.675	-0.922	0.426	-1.213	0.604	-0.594	29
GM 7	0.071	-0.457	-0.348	0.306	0.815	0.397	0.066	0.019	17
GM 8	1.457	0.238	-0.045	0.223	0.820	-0.848	-0.490	0.489	5
GM 9	-0.180	-1.203	-0.385	-0.535	0.072	-0.560	-0.510	-0.491	27
GM 10	0.117	0.104	0.560	0.324	1.766	1.693	-0.327	0.443	7
GM 11	0.314	-0.853	-0.072	-0.983	0.051	0.303	-0.196	-0.203	23
GM 12	-0.052	-0.843	-0.435	0.340	1.199	-0.309	-0.168	-0.135	21
GM 13	-0.261	-0.434	-0.209	-1.185	0.064	-0.932	0.720	-0.361	26
GM 14	-0.538	-0.446	-0.938	-0.360	-1.124	0.697	-0.364	-0.514	28
GM 15	0.004	-0.387	-0.603	1.116	0.579	-0.282	1.587	0.097	12
GM 16	-0.039	-2.032	-1.686	0.383	0.169	0.095	-1.370	-0.700	30
GM 17	0.156	-0.954	1.270	1.854	-0.184	2.059	1.354	0.472	6
GM 18	0.092	-0.425	1.453	-1.250	-0.726	0.954	0.454	0.028	15
GM 19	-2.583	0.964	2.507	1.477	-0.147	-0.986	-1.505	-0.181	22
GM 20	-1.395	0.605	-2.228	-1.322	-0.318	0.533	-0.209	-0.786	32
GM 21	-1.326	0.151	-0.122	-0.988	-1.666	-0.172	-0.668	-0.708	31
GM 22	0.639	0.983	-0.970	1.072	-0.878	-1.299	-1.975	0.091	13
GM 23	-0.933	-0.455	0.906	-0.031	-0.713	-0.888	0.838	-0.318	25
GM 24	-0.802	0.587	0.321	-1.407	1.942	-0.530	0.388	-0.073	20
GM 25	-0.711	0.933	-0.053	1.089	-0.623	0.008	-0.508	0.023	16
GM 26	-0.529	0.445	-1.503	0.414	-0.277	1.633	1.529	-0.056	19
GM 27	-0.453	0.314	0.313	1.046	0.703	-1.419	0.493	0.102	11
GM 28	-0.379	2.069	-0.357	-0.535	-0.360	0.878	0.116	0.236	10
GM 29	1.152	0.972	-0.488	1.447	-0.314	-1.049	0.119	0.554	3
GM 30	0.641	0.678	1.142	-0.925	-1.351	2.249	-1.495	0.330	8
GM 31	1.658	1.335	0.398	-0.187	-0.903	-0.446	1.631	0.789	2
GM 32	2.552	1.145	-0.658	0.305	0.087	0.075	-0.834	0.897	1

## Discussion

*Castanea mollissima* (chestnut) is a cross-pollinated plant with a wide distribution range and abundant germplasm resources formed during its long-term evolution [8]. Affected by their own genetic factors and the environment, different superior plants of chestnut differ in fruit phenotype and quality traits [7]. The development prospects of Wangmo *C*. *mollissima* are great, but its development and utilization are still in their infancy. The analysis and scientific evaluation of the fruit phenotypic and quality traits of Wangmo *C*. *mollissima* provide a basis for the rational utilization of chestnut resources [26]. Many plant traits are correlated. Before the fruit phenotypic and quality traits of Wangmo *C*. *mollissima* are scientifically evaluated, they can be assessed on the basis of correlation analysis of these traits, which can intuitively reflect the relationships between them. Correlation analysis is applied to determine the degree of correlation between two or more correlated variables [27]. In this study, 15 and 16 pairs of fruit phenotypic and quality traits showed extremely significant positive correlations (P<0.01) and significant positive correlations (P<0.05), respectively, and 4 and 15 pairs showed extremely significant negative correlations (P<0.01) and significant negative correlations (P<0.05), respectively. These results indicated relatively strong relationships between the quality indicators of Wangmo *C*. *mollissima* and suggested that the value of one indicator may be affected by that of other indicators. In addition, 10 indicators of fruit phenotype and 13 indicators of quality did not show significant correlations, indicating that the correlations between chestnut fruit phenotypic and quality traits were relatively independent, similar to the research results of Jiang [9]. In the breeding and cultivation of superior Wangmo *C*. *mollissima* plants, it is necessary to improve certain traits; however, plant traits are relatively stable, and the improvement potential is small. Whether a trait can be improved indirectly on the basis of its degree of correlation with other traits needs to be further studied in detail. Liu et al. [28,29] found that the CVs of morphological indices such as the nut height, width and thickness of chestnut farm varieties and hybrid offspring were all less than 15.0%, while the CV of single-grain weight was large, reaching 26.6%. Jiang [9,30] found that the average CV of nut quality traits was greater than the average CV of nut phenotypic traits, so the quality traits of chestnut fruits exhibited greater variation than the phenotypic traits. The results revealed different degrees of variation in the fruit phenotypic and quality traits of 32 superior Wangmo *C*. *mollissima* plants. The CV values ranged from 7.61% to 48.86%. The CV for fruit phenotypic traits was 16.01%, while for fruit quality traits, it was 20.32%. This indicates that the variation in the fruit quality traits of Wangmo *C*. *mollissima* was more extensive than that in the phenotypic traits. Consequently, the potential for improvement in phenotypic traits was greater. The results of this study are consistent with the results of previous studies, indicating that fruit morphological traits are more stable than fruit quality traits.

The single weight of a chestnut bract has an important influence on chestnut yield, and the moisture content of the nuts has a great influence on storage [31]. By analyzing the phenotypic traits of 32 Wangmo *C*. *mollissima* fruits, it was found that there were significant differences in the single weight and moisture content of chestnut bracts among different superior strains of chestnut. According to the results of cluster analysis, germplasms of different sizes can be screened to meet the diversified needs of the market [32,33]. The soluble sugar content can directly affect the sweetness and taste of chestnut, and the stress resistance of plants is closely related to soluble sugar and starch [34]. Starch is the main factor affecting the edible quality of chestnut, affecting the taste and quality of processed chestnut food products [35]. Protein is the only nitrogen source in the human body, and its content directly affects the emulsification and water absorption of *C*. *mollissima* [36]. The quality of *C*. *mollissima* is directly reflected in the contents of soluble sugar, starch and soluble protein. The results showed that the soluble sugar content of Wangmo *C*. *mollissima* was 13.07%, the starch content was 53.93%, and the soluble protein content was 0.37%. Previous studies revealed that the soluble sugar content of *C*. *mollissima* in the northern mountainous area of Yanshan was 9.80%, the starch content was 34.3%, and the soluble protein content was 0.56% [8]. The soluble sugar content of Chinese farm cultivars was 8.91%, the starch content was 49.70%, and the soluble protein content was 4.99% [28]. The soluble sugar content of the main cultivar in the middle and lower reaches of the Yangtze River was 5.68%, the starch content was 66.27%, and the soluble protein content was 6.14% [10]. The soluble sugar content of 90 local chestnut cultivars was 4.39–23.33%, and the starch content was 14.58–61.61% [30]. Overall, the soluble sugar content of Wangmo *C*. *mollissima* is lower than those of only the main varieties in the middle and lower reaches of the Yangtze River and Shaanxi Wuming No. 1 among the 90 local cultivars in China, and its soluble protein content is the lowest. Compared with that of other cultivars, the starch content of Wangmo *C*. *mollissima* is relatively high, the soluble sugar content is intermediate, and the soluble protein content is relatively low. Correlation analysis showed that the moisture content of nuts was significantly negatively correlated with Ca, soluble protein, soluble sugar and fat; the moisture content of Wangmo *C*. *mollissima* nuts was 65.47%, which is much higher than those of other cultivars of chestnut. A low moisture content in nuts not only enables them to be more resistant to storage but also has a strong impact on their quality. According to determinations by Chinese institutional authorities, the K content of Wangmo *C*. *mollissima* ranks first in China, but there have been few studies on the mineral content of chestnut fruits in different production areas in China and other countries. The K content of the 25 main chestnut cultivars and 5 representative northern chestnut cultivars in the middle and lower reaches of the Yangtze River is reportedly between 5060 mg/kg and 6568 mg/kg, with an average of 5815 mg/kg [9], while the K content of Wangmo *C*. *mollissima* in this study was 4948–9271 mg/kg, with an average of 7652 mg/kg. The K content is much higher than that of the other 30 chestnut cultivars. These research results provide data support for Wangmo *C*. *mollissima*, which has the highest K content in China. The K content of GM-8, GM-23 and GM-28 exceeded 9000 mg/kg, indicating that they can be cultivated and promoted as high-quality varieties with a high K content. The growth of *C*. *mollissima* is regulated by multiple factors. The climate of Wangmo in Guizhou is complex. The contents of soluble protein and soluble sugar in Wangmo *C*. *mollissima* are low, and the contents of K and starch are high. The correlation between traits can be used to increase the content of soluble protein and soluble sugar by reducing the moisture content of nuts, but this phenomenon is shaped by many factors. In future research, the potential factors need to be further evaluated, and systematic investigation and research are needed.

In this study, the combination of clustering analysis and PCA was used to comprehensively evaluate Wangmo *C*. *mollissima*. The main purpose was to explain most of the information with fewer variables and simplify the complexity of the data [14]. Cluster analysis supports a quantitative determination of the relationships between samples based on the correlations between different characteristics or the similarity or difference of indicators between samples. Cluster analysis is a relatively simple analysis and evaluation method for classifying and studying the differences in species cultivar resources. The more traits that are included in the cluster analysis, the more comprehensively they can reflect the cultivar (species). In this study, hierarchical clustering analysis was carried out according to Euclidean genetic distance. The 32 superior Wangmo *C*. *mollissima* plants were clustered into three categories. On the one hand, excellent germplasm resources of Wangmo *C*. *mollissima* can be exploited. On the other hand, the large genetic differences between different genotypes can be used for cross-breeding to produce extensive variation, and it would be easy to select super-parents and adaptable strains [37]. Cluster analysis also revealed that most of the individuals from the same source were clustered together, indicating that the multidirectionality of genetic divergence was positively associated with the geographical distribution, but there were also some individuals with low genetic divergence and a small geographical distribution. Individuals from different provenances may also often cluster in the same group, while individuals from the same source may cluster in different groups. This may be the result of natural selection that determines the multidirectionality of genetic divergence among individuals [38]. Therefore, the selection of materials between groups with large genetic distances as hybrid parents often yields better results.

Scientific evaluation is the basis for screening germplasm resources. Numerous researchers have used the fuzzy evaluation method [39], the equidistant grading evaluation method [40] and PCA [41] to evaluate germplasm resources. The fuzzy evaluation method and equidistant grading evaluation method have advantages but are limited because of artificial intervention (expert scoring). Multiple factors lead to uncertainty in the evaluation results. These methods are applicable when there are few evaluated trait indicators and the main indicators can be selected as highlights. In this study, PCA categorized similar factors into a representative common factor through dimension reduction, thereby reducing the number of indicators. Then, a comprehensive score was calculated based on the common factor scores and variance contribution ratios. However, by using this method to convert multiple indicators into a small number of indicators for quality evaluation, the interaction and influence between multiple indicators are not comprehensively considered, which may lead to a loss of information. Therefore, we combined the unilateral influence of factors and the mutual influence of factors through ANOVA and correlation analysis. The use of this combination for the analysis of chestnut quality can balance multiple aspects, reduce the limitations of a single method, and enhance the reliability of the obtained results. In addition, this combined method is suitable for screening large samples of germplasm resources and improving the comprehensive evaluation of chestnut quality.

China has excellent chestnut germplasm resources; however, for a long time, the breeding goals for chestnut fruit quality have mainly focused on high yield, nutrition, and aroma [10,28,42,43]. To date, the sensory factor characteristics of chestnut have not been clarified, and research on sensory factor traits has been neglected. For this reason, resource advantages have not been fully utilized. Consequently, sensory factors were not considered in any part of the evaluation system in this study when selecting evaluation factors. In future research, sensory factors should be considered for chestnuts. The number of samples should be expanded to explore the quality evaluation of chestnuts in a more detailed and comprehensive manner. In addition, molecular technology can be used to further analyze the reasons for quality differences between chestnuts. This study mainly focused on the fruit traits of Wangmo chestnut, without considering the growth status of the tree. Research has revealed that the growth status of trees can affect the morphology and economic characteristics of their fruits [44], although there is still a lack of strong evidence to explain the mechanism underlying this phenomenon. Nevertheless, the breeding of improved cultivars directly affects the economic value of the seeds of economic forests, which may have an enormous economic impact. Therefore, attention should also be given to this factor, and confirmatory research should be carried out in the field of economic forest cultivation.

In this study, the PCA method was used to comprehensively and objectively evaluate many fruit traits. On the basis of PCA, the top ten superior plants based on comprehensive quality were GM32, GM31, GM29, GM1, GM8, GM17, GM10, GM30, GM3 and GM28, which can be used as alternatives to develop excellent germplasm resources. The findings of this study lay a theoretical foundation for selecting chestnut cultivars with excellent fruit quality and provide a reference for screening excellent chestnut germplasm resources, which is of great significance in solving the current problem of the lack of high-quality chestnut varieties. However, follow-up observations and experiments are still needed to study their stress resistance and yield stability.
